# Supplementary material for: Crystal Structure of Pyrrolysyl-tRNA Synthetase from a Methanogenic Archaeon ISO4-G1 and Its Structure-Based Engineering for Highly-Productive Cell-Free Genetic Code Expansion with Non-Canonical Amino Acids
Source: Int J Mol Sci. 2023 Mar 26;24(7):6256. doi: 10.3390/ijms24076256 (PMC10094482; doi:10.3390/ijms24076256)
Supplement: Supplementary file 1 [file ijms-24-06256-s001.zip › ijms-2268100-supplementary.pdf]

Fig. S1 Yanagisawa *et al.*

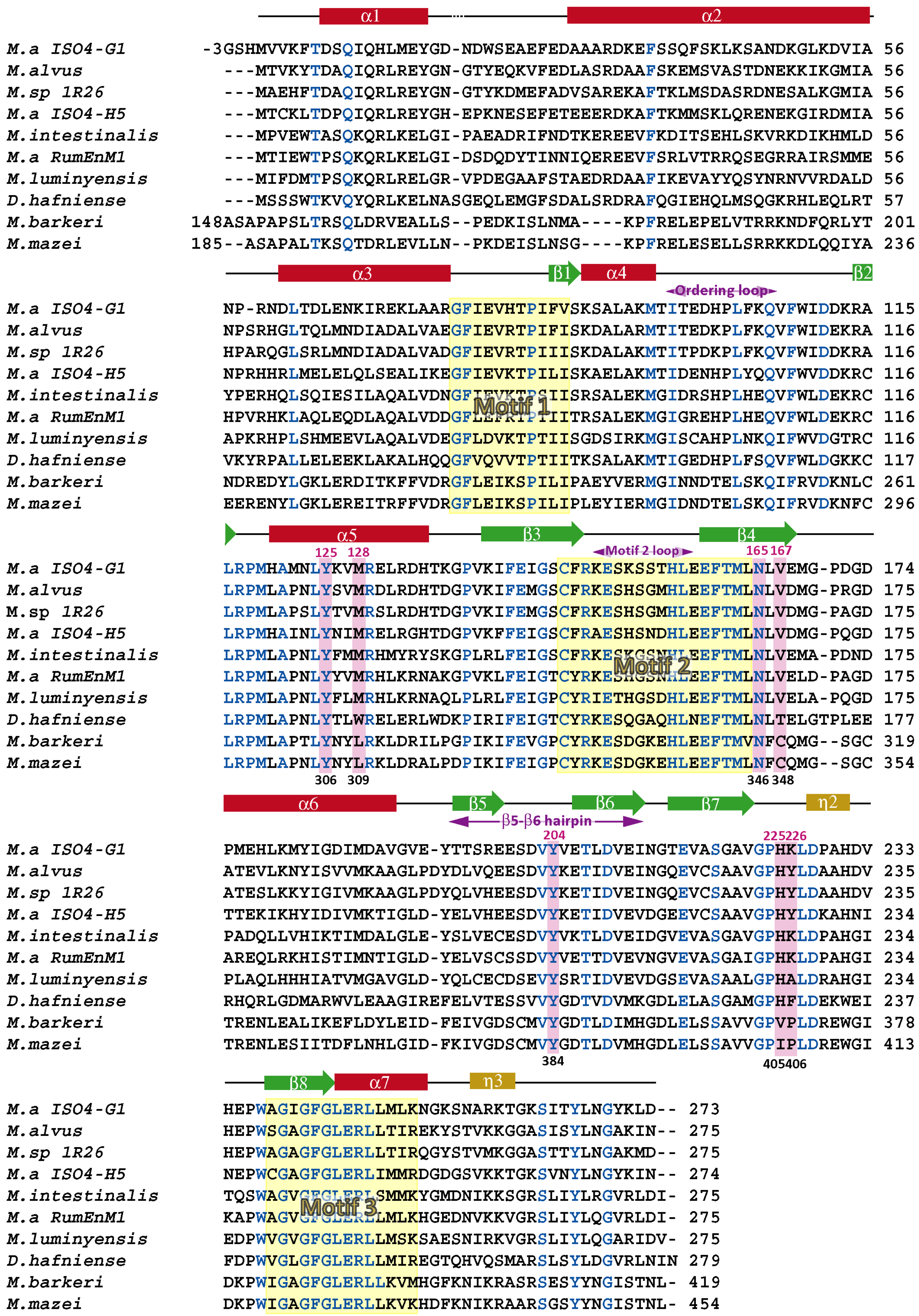

Fig. S1. Structure-based sequence alignments of ISO4-G1 PylRS and other PylRSs. The PylRS sequences were aligned with the program CLUSTAL W [89], and then parts of the alignments were adjusted manually. Highly conserved residues among the PylRSs are shown in blue. The secondary structures (α-helices, 3_{10} helices, and β-sheets) are shown as wine red bars, olive bars, and green arrows, respectively, above the sequence alignments. Numbers at the top and bottom correspond to the amino acid residues of ISO4-G1 PylRS and *Mm*PylRS, respectively. Dashes represent breaks in the actual amino acid sequences to allow sequence alignments with PylRSs. Motifs 1, 2, and 3 are colored yellow. The ordering loop, the motif-2 loop, and the β5-β6 hairpin are shown on the top line. Amino acid residues that were mutated in this study are colored pink. Accession numbers are as follows. ISO4-G1 PylRS (*M.a* ISO4-G1, AMK13702); *Ma*PylRS (*M. alvus*, WP_015505008); 1R26PylRS (*M. sp 1R26*, WP_058747239); ISO4-H5 PylRS (*M.a* ISO4-H5, WP_066075773); RumPylRS (*M.a RumEnM1*, KQM11560); *Ml*PylRS (*M. luminyensis*, WP_019176308); *Mt*PylRS (*M. termitum*, WP_048111907); *Mi*PylRS (*M. intestinalis*, WP_020448777); *Dh*PylSc (*D. hafniense*, WP_018307530); *Mb*PylRS (*M. barkeri*, Q6WRH6); and *Mm*PylRS (*M. mazei*, Q8PWY1).

Fig. S2 Yanagisawa *et al.*

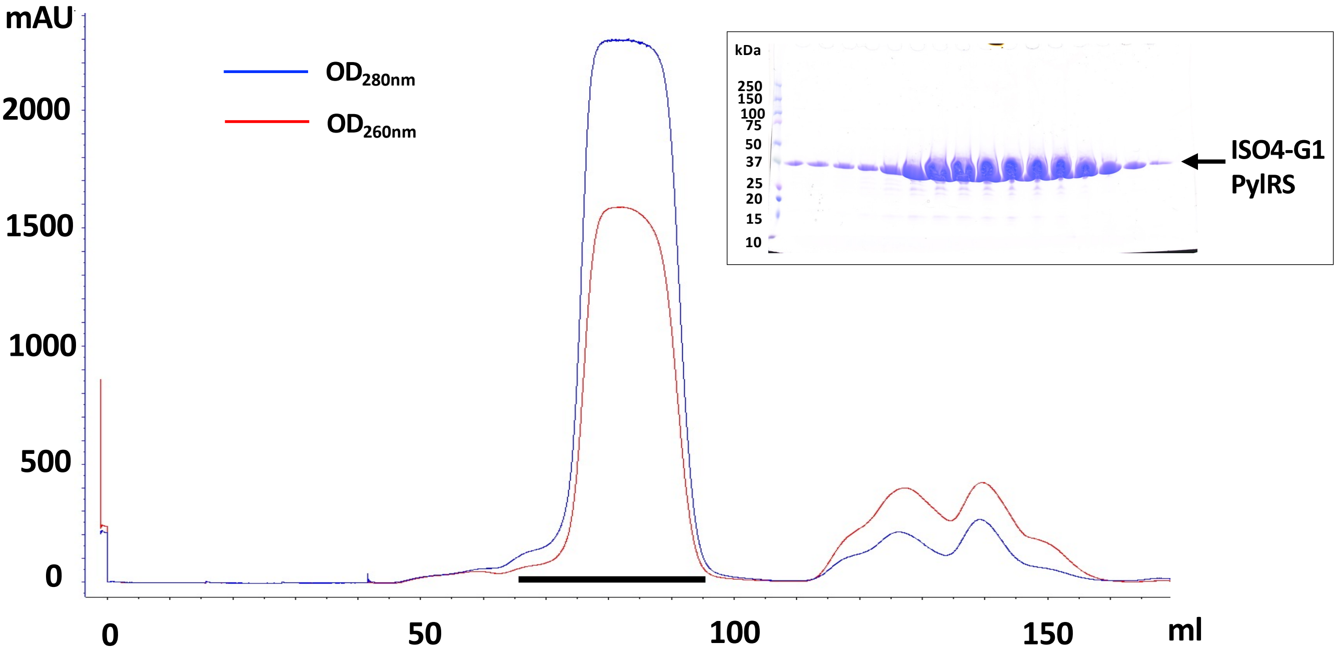

Fig. S2. Chromatogram for the purification of ISO4-G1 PylRS by Superdex 200 size-exclusion chromatography. SDS-PAGE analysis of the ISO4-G1 PylRS fractions (inset). The absorbances of the ISO4-G1 PylRS fractions are saturated at 280 nm and 260 nm. The black bar represents the range of fractions subjected to SDS-PAGE analysis.

Fig.S3 Yanagisawa *et al.*

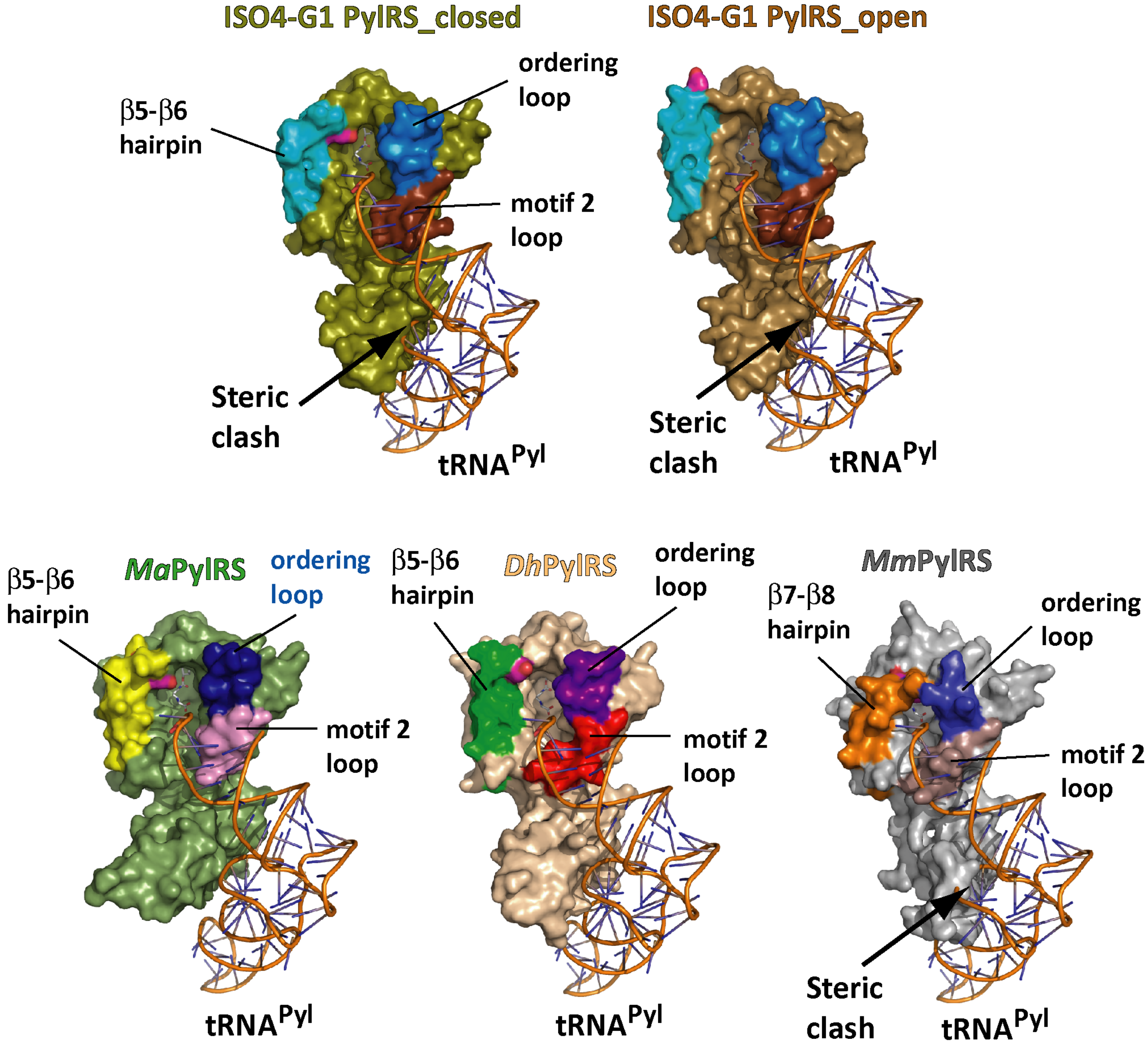

Fig. S3. Structural comparison of ISO4-G1 PylRS with *Ma*PylRS, *Mm*PylRSc, and *Dh*PylSc•tRNA^{Pyl}.

Superimpositions of the ISO4-G1 PylRS with the *Ma*PylRS, the *Dh*PylSc•tRNA^{Pyl} complex (PDB code: 2ZNI), the apo form (PDB: 2E3C), and the Pyl-AMP-bound *Mm*PylRSc (PDB: 2ZIM) structures, represented by surface models. The ordering loop, the motif-2 loop, and the β5-β6 hairpin (β7-β8 hairpin in *Mm*PylRS) are colored differently. The catalytic core structures of ISO4-G1 PylRS, *Ma*PylRS, *Dh*PylSc, and *Mm*PylRSc superimposed well, but the two α-helices (α1 and α2) of *Mm*PylRSc are slightly tilted and cause steric hindrance with tRNA^{Pyl}.

Fig. S4 Yanagisawa *et al.*

a Open (molecule A)

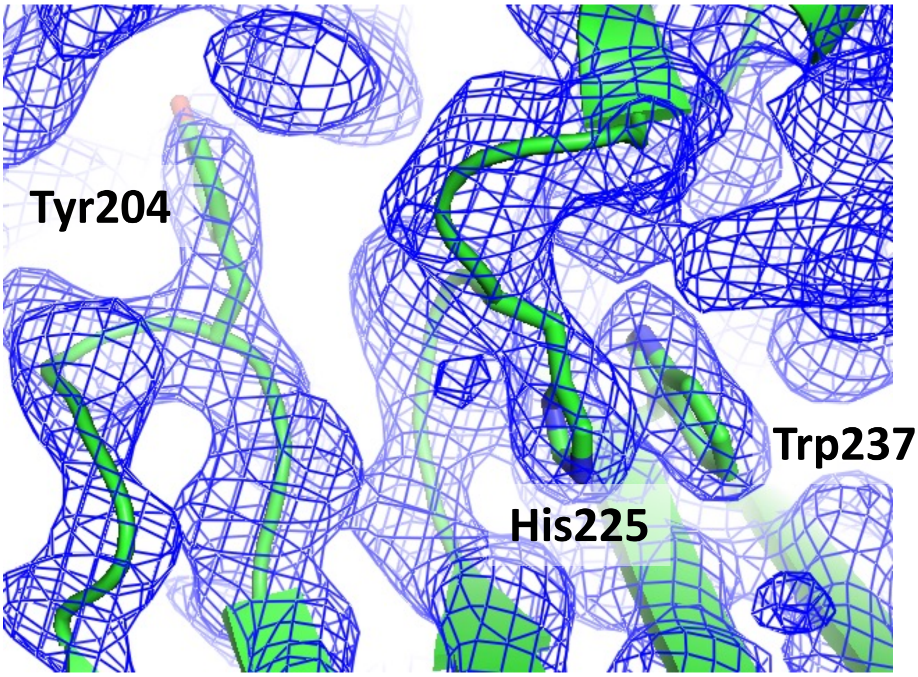

b Closed (molecule B)

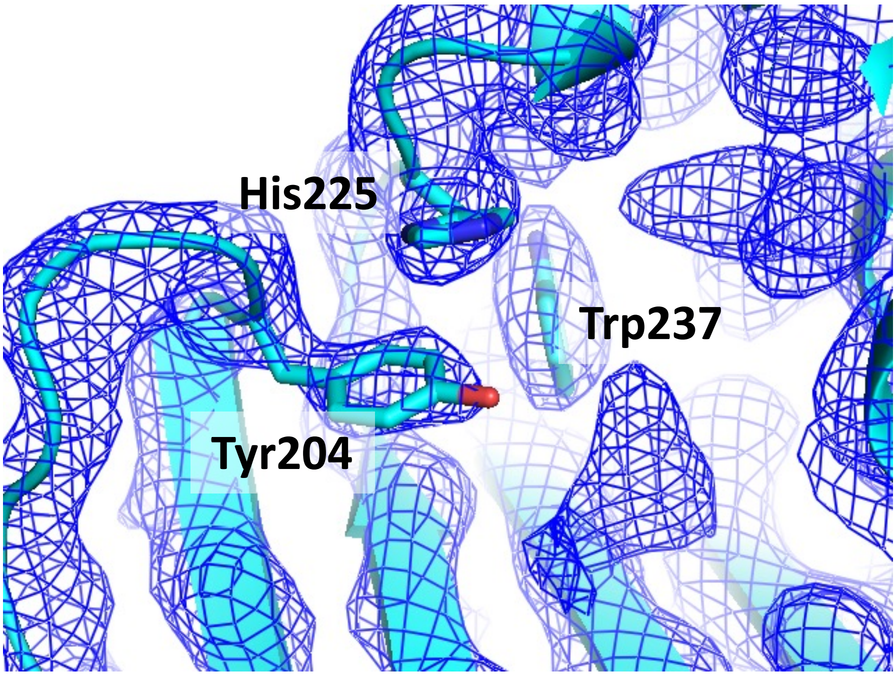

Fig. S4. Electron density map of the β5-β6 region in the ISO4-G1 PylRS structure. The 2*Fo-Fc* electron density map for the regions around Tyr204, His225, and Trp237 is represented as a blue mesh at a contour level of 1σ. (a) The open conformation. (b) The closed conformation. The Tyr204, His225, and Trp237 residues are shown as stick models.

Fig. S5 Yanagisawa *et al.*

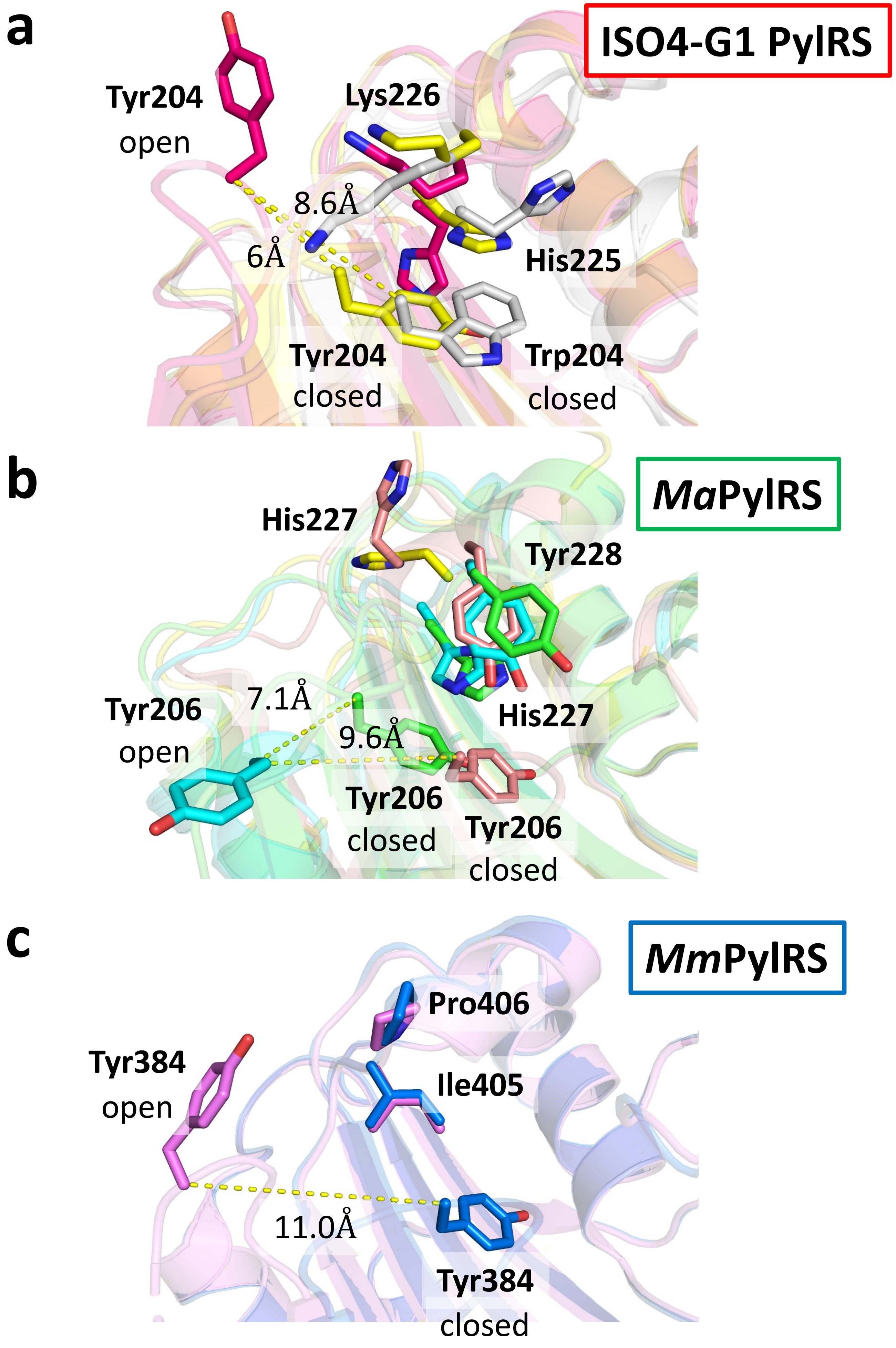

Fig. S5. Conformational changes of the active-site residues in the open and closed forms of the ISO4-G1 PylRS, *Ma*PylRS, and *Mm*PylRS structures. (a) The open and closed conformations of the ISO4-G1 PylRS apo form (magenta and yellow, respectively), and the closed conformation of the ISO4-G1 PylRS mutant (7R6O, white). (b) The open and closed conformations of the *Ma*PylRS apo form (6JP2, cyan and light green, respectively), and the closed conformation of the *de novo* screened *Ma*PylRS(N166A/C168G/W239C) mutant bound to acrydonylalanine and AMPPNP (8DQG, vermilion). Tyr206 is disordered in the AMPPNP-bound form (8DQG, yellow). (c) The open conformation of the *Mm*PylRS apo form (pink), and the closed conformation of *Mm*PylRS bound to pyrrolysyladenylate (2Q7H, sky blue). The translucent ribbon models are shown in the background. The ISO4-G1 PylRS Tyr205 residue corresponds to Tyr206 in *Ma*PylRS, and to Tyr384 in *Mm*PylRS. The ISO4-G1 PylRS His225 residue corresponds to His227 in *Ma*PylRS, and to Ile405 in *Mm*PylRS.The ISO4-G1 PylRS Lys226 residue corresponds to Tyr228 in *Ma*PylRS, and to Pro406 in *Mm*PylRS. Each residue is shown as a stick model.

Fig.S6 Yanagisawa *et al.*

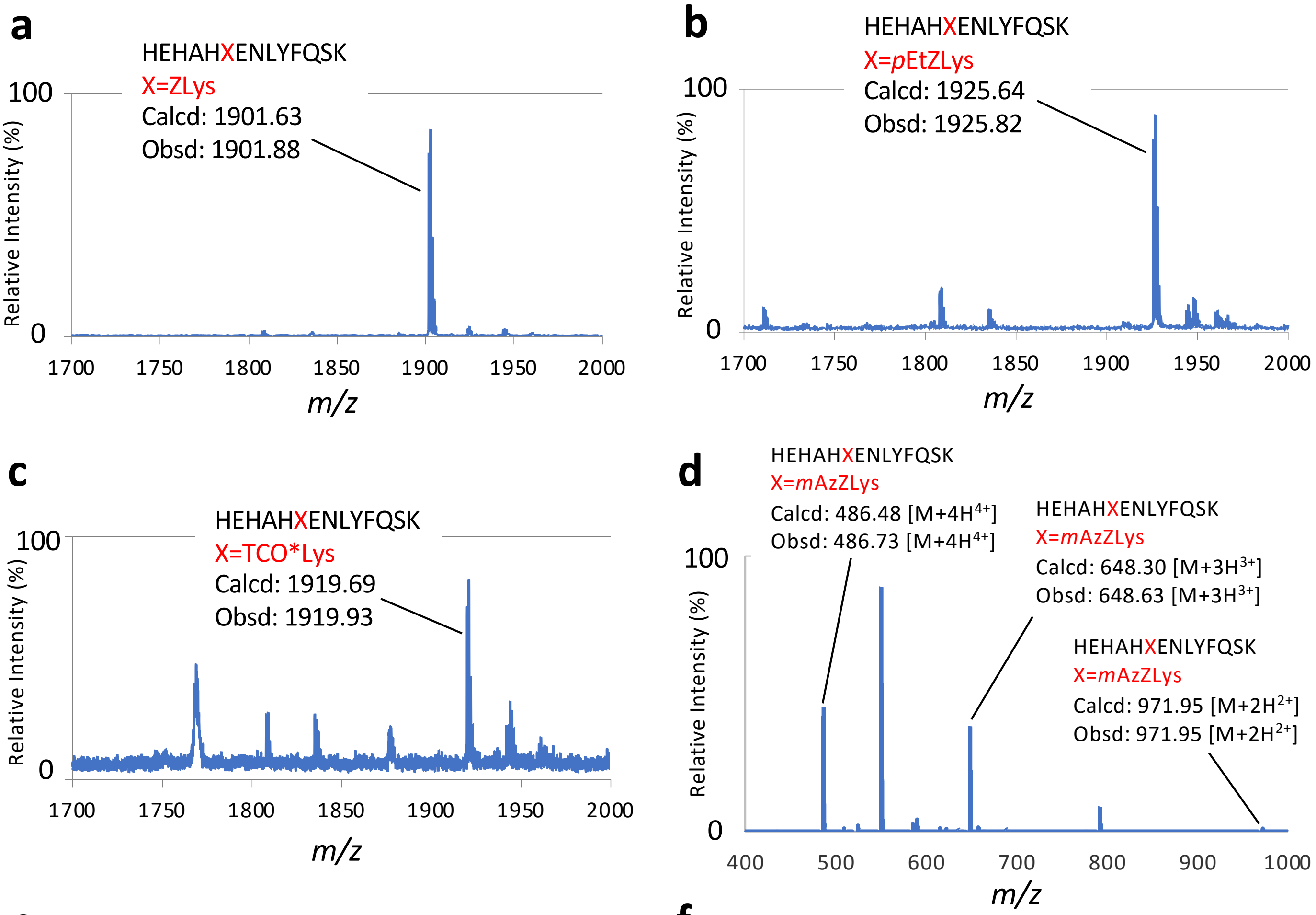

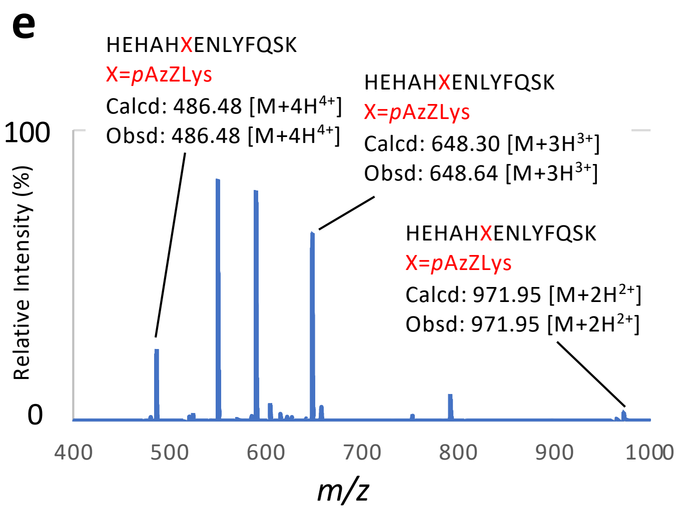

f

N11-17amber-GFPS1 sequence
MKDHLIHNHHKHEHAHXENLYFQSKGEELFTGVVPIL
VELDGDVNGHKFSVSGEGEGDATYGKLTLKFICTTGK
LPVPWPTLVTTLTYGVQCFSRYPDHMKRHDFFKSAM
PEGYVQERTISFKDDGNYKTRAEVKFEGDTLVNRIELK
GIDFKEDGNILGHKLEYNYNSHNVYITADKQKNGIKA
NFKIRHNIEDGSVQLADHYQQNTPIGDGPVLLPDNH
YLSTQSALSKDPNEKRDHMVLLEFVTAAGITHGMDE
LYK

Fig. S6. Mass spectrometry analysis of N11-GFPS1 proteins containing non-canonical amino acids. The amino acid sequence of GFPS1, with a 24-residue N11-peptide tag at the N-terminus, is shown in (f). The codon of the N11-GFPS1 residue Ala17, which is highlighted by a red X, is mutated to an amber (UAG) codon. The incorporations of ZLys (a), *p*EtZLys (b), and TCO*Lys (c), at position 17 in N11-GFPS1, were confirmed by MALDI-TOF analyses. The PMF analysis of the tryptic digests by MALDI-TOF mass spectrometry revealed major peaks (obsd.: *m*/*z* 1,901.88 $[M+H]^+$, *m*/*z* 1,925.82 $[M+H]^+$, *m*/*z* 1,919.93 $[M+H]^+$) that match the theoretical masses of the tryptic peptides HEHAHXENLYFQSK, where X represents ZLys, *p*EtZLys, and TCO*Lys, respectively (calcd.: *m*/*z* 1,901.63 $[M+H]^+$, *m*/*z* 1,925.64 $[M+H]^+$, *m*/*z* 1,919.69 $[M+H]^+$). The incorporations of *m*AzZLys (d) and *p*AzZLys (e), at position 17 in N11-GFPS1, were confirmed by ESI-MS analyses of the tryptic peptide HEHAHXENLYFQSK (X represents a non-canonical amino acid). The ESI mass analysis revealed the tryptic peptides containing *m*AzZLys (obsd.: *m*/*z* 971.95 $[M+2H]^{2+}$, calcd.: *m*/*z* 971.95 $[M+2H]^{2+}$; obsd.: *m*/*z* 648.63 $[M+3H]^{3+}$, calcd.: *m*/*z* 648.30 $[M+3H]^{3+}$; obsd.: *m*/*z* 486.73 $[M+4H]^{4+}$, calcd.: *m*/*z* 486.48 $[M+4H]^{4+}$) and *p*AzZLys (obsd.: *m*/*z* 971.95 $[M+2H]^{2+}$, calcd.: *m*/*z* 971.95 $[M+2H]^{2+}$; obsd.: *m*/*z* 648.64 $[M+3H]^{3+}$, calcd.: *m*/*z* 648.30 $[M+3H]^{3+}$; obsd.: *m*/*z* 486.48 $[M+4H]^{4+}$, calcd.: *m*/*z* 486.48 $[M+4H]^{4+}$). The observed molecular masses agreed well with the calculated masses.

Table S1

Data collection and refinement statistics.

	ISO4-G1 PylRS
PDB code	8IFJ
X-ray source	SPring-8<br>BL32XU
No. of crystals	1
Wavelength	1.0000
Space group	$P2_12_12_1$
Cell dimensions<br>*a* (Å)<br>*b* (Å)<br>*c* (Å)	 98.51<br>102.68<br>349.86
α, β, γ (°)	90, 90, 90
Resolution (Å)	50−2.78 (2.85−2.78)
I/σ (I)	14.47 (1.32)
Completeness (%)	99.73 (99.77)
No. reflections	90,164
Redundancy (%)	5.99 (6.07)
$^aR_{meas}$	0.16 (1.94)
Refinement	
$^bR_{work}$ / $^cR_{free}$ (%)	23.3/29.5
Resolution (Å)	49.9−2.78
No. atoms<br>protein<br>water	 21,566<br>49
No. reflections<br>(total / test)	 90,021/1,999
Average B-factors<br>protein<br>water	 100.10<br>57.18
R.m.s. deviations<br>Bond length (Å)<br>Bond angles (°)	 0.004<br>0.640
Ramachandran plot<br>Most favored (%)<br>Allowed (%)<br>Disallowed (%)	 96.09<br>3.91<br>0.00

The numbers in parentheses are for the last shell.

[a] $R_{meas} = S_{hkl}(n^{1/2/}(n\text{-}1)^{1/2})S_i |I_{avg}\text{-}I_i| / S_{hkl} SI_i$.

[b] $R_{work} = S_{hkl} |F_o\text{-}F_c| / S_{hkl} F_o$ for reflections of work set.

[c] $R_{free} = S_{hkl} |F_o\text{-}F_c| / S_{hkl} F_o$ for reflections of test set [2.2% of total reflections for ISO4-G1 PylRS].

Table S2

DNA sequence of the pET28_ISO4-G1 PylRS(Y125A/M128L) plasmid.

The ISO4-G1 PylRS(Y125A/M128L) gene, shown in red capital letters, was inserted into the *Nde*I and *Bam*HI sites of the pET28 vector. The 125Ala (GCT) and 128Leu (CTG) codons are highlighted in cyan.

```
tggcgaatgggacgcgccctgtagcggcgcattaagcgcggcgggtgtggtggttacgcgcagcgtgacc
gctacacttgccagcgccctagcgcccgctcctttcgctttcttcccttcctttctcgccacgttcgccg
gctttccccgtcaagctctaaatcgggggctccctttagggttccgatttagtgctttacggcacctcga
ccccaaaaaacttgattagggtgatggttcacgtagtgggccatcgccctgatagacggtttttcgccct
ttgacgttggagtccacgttctttaatagtggactcttgttccaaactggaacaacactcaaccctatct
cggtctattcttttgatttataagggattttgccgatttcggcctattggttaaaaaatgagctgattta
acaaaaatttaacgcgaattttaacaaaatattaacgtttacaatttcaggtggcacttttcggggaaat
gtgcgcggaacccctatttgtttatttttctaaatacattcaaatatgtatccgctcatgaattaattct
tagaaaaactcatcgagcatcaaatgaaactgcaatttattcatatcaggattatcaataccatattttt
gaaaaagccgtttctgtaatgaaggagaaaactcaccgaggcagttccataggatggcaagatcctggta
tcggtctgcgattccgactcgtccaacatcaatacaacctattaatttcccctcgtcaaaaataaggtta
tcaagtgagaaatcaccatgagtgacgactgaatccggtgagaatggcaaaagtttatgcatttctttcc
agacttgttcaacaggccagccattacgctcgtcatcaaaatcactcgcatcaaccaaaccgttattcat
tcgtgattgcgcctgagcgagacgaaatacgcgatcgctgttaaaaggacaattacaaacaggaatcgaa
tgcaaccggcgcaggaacactgccagcgcatcaacaatattttcacctgaatcaggatattcttctaata
cctggaatgctgttttcccggggatcgcagtggtgagtaaccatgcatcatcaggagtacggataaaatg
cttgatggtcggaagaggcataaattccgtcagccagtttagtctgaccatctcatctgtaacatcattg
gcaacgctacctttgccatgtttcagaaacaactctggcgcatcgggcttcccatacaatcgatagattg
tcgcacctgattgcccgacattatcgcgagcccatttatacccatataaatcagcatccatgttggaatt
taatcgcggcctagagcaagacgtttcccgttgaatatggctcataacaccccttgtattactgtttatg
taagcagacagttttattgttcatgaccaaaatcccttaacgtgagttttcgttccactgagcgtcagac
cccgtagaaaagatcaaaggatcttcttgagatcctttttttctgcgcgtaatctgctgcttgcaaacaa
aaaaaccaccgctaccagcggtggtttgtttgccggatcaagagctaccaactctttttccgaaggtaac
tggcttcagcagagcgcagataccaaatactgtccttctagtgtagccgtagttaggccaccacttcaag
aactctgtagcaccgcctacatacctcgctctgctaatcctgttaccagtggctgctgccagtggcgata
agtcgtgtcttaccgggttggactcaagacgatagttaccggataaggcgcagcggtcgggctgaacggg
gggttcgtgcacacagcccagcttggagcgaacgacctacaccgaactgagatacctacagcgtgagcta
tgagaaagcgccacgcttcccgaagggagaaaggcggacaggtatccggtaagcggcagggtcggaacag
gagagcgcacgagggagcttccagggggaaacgcctggtatctttatagtcctgtcgggtttcgccacct
ctgacttgagcgtcgatttttgtgatgctcgtcaggggggcggagcctatggaaaaacgccagcaacgcg
gcctttttacggttcctggccttttgctggccttttgctcacatgttctttcctgcgttatcccctgatt
ctgtggataaccgtattaccgcctttgagtgagctgataccgctcgccgcagccgaacgaccgagcgcag
cgagtcagtgagcgaggaagcggaagagcgcctgatgcggtattttctccttacgcatctgtgcggtatt
tcacaccgcatatatggtgcactctcagtacaatctgctctgatgccgcatagttaagccagtatacact
ccgctatcgctacgtgactgggtcatggctgcgccccgacacccgccaacacccgctgacgcgccctgac
gggcttgtctgctcccggcatccgcttacagacaagctgtgaccgtctccgggagctgcatgtgtcagag
gttttcaccgtcatcaccgaaacgcgcgaggcagctgcggtaaagctcatcagcgtggtcgtgaagcgat
tcacagatgtctgcctgttcatccgcgtccagctcgttgagtttctccagaagcgttaatgtctggcttc
tgataaagcgggccatgttaagggcggttttttcctgtttggtcactgatgcctccgtgtaagggggatt
tctgttcatgggggtaatgataccgatgaaacgagagaggatgctcacgatacgggttactgatgatgaa
catgcccggttactggaacgttgtgagggtaaacaactggcggtatggatgcggcgggaccagagaaaaa
tcactcagggtcaatgccagcgcttcgttaatacagatgtaggtgttccacagggtagccagcagcatcc
tgcgatgcagatccggaacataatggtgcagggcgctgacttccgcgtttccagactttacgaaacacgg
aaaccgaagaccattcatgttgttgctcaggtcgcagacgttttgcagcagcagtcgcttcacgttcgct
cgcgtatcggtgattcattctgctaaccagtaaggcaaccccgccagcctagccgggtcctcaacgacag
gagcacgatcatgcgcacccgtggggccgccatgccggcgataatggcctgcttctcgccgaaacgtttg
gtggcgggaccagtgacgaaggcttgagcgagggcgtgcaagattccgaataccgcaagcgacaggccga
```

tcatcgtcgcgctccagcgaaagcggtcctcgccgaaaatgacccagagcgctgccggcacctgtcctac
gagttgcatgataaagaagacagtcataagtgcggcgacgatagtcatgccccgcgcccaccggaaggag
ctgactgggttgaaggctctcaagggcatcggtcgagatcccggtgcctaatgagtgagctaacttacat
taattgcgttgcgctcactgcccgctttccagtcgggaaacctgtcgtgccagctgcattaatgaatcgg
ccaacgcgcggggagaggcggtttgcgtattgggcgccagggtggtttttcttttcaccagtgagacggg
caacagctgattgcccttcaccgcctggccctgagagagttgcagcaagcggtccacgctggtttgcccc
agcaggcgaaaatcctgtttgatggtggttaacggcgggatataacatgagctgtcttcggtatcgtcgt
atcccactaccgagatatccgcaccaacgcgcagcccggactcggtaatggcgcgcattgcgcccagcgc
catctgatcgttggcaaccagcatcgcagtgggaacgatgccctcattcagcatttgcatggtttgttga
aaaccggacatggcactccagtcgccttcccgttccgctatcggctgaatttgattgcgagtgagatatt
tatgccagccagccagacgcagacgcgccgagacagaacttaatgggcccgctaacagcgcgatttgctg
gtgacccaatgcgaccagatgctccacgcccagtcgcgtaccgtcttcatgggagaaaataatactgttg
atgggtgtctggtcagagacatcaagaaataacgccggaacattagtgcaggcagcttccacagcaatgg
catcctggtcatccagcggatagttaatgatcagcccactgacgcgttgcgcgagaagattgtgcaccgc
cgctttacaggcttcgacgccgcttcgttctaccatcgacaccaccacgctggcacccagttgatcggcg
cgagatttaatcgccgcgacaatttgcgacggcgcgtgcagggccagactggaggtggcaacgccaatca
gcaacgactgtttgcccgccagttgttgtgccacgcggttgggaatgtaattcagctccgccatcgccgc
ttccactttttcccgcgttttcgcagaaacgtggctggcctggttcaccacgcgggaaacggtctgataa
gagacaccggcatactctgcgacatcgtataacgttactggtttcacattcaccaccctgaattgactct
cttccgggcgctatcatgccataccgcgaaaggttttgcgccattcgatggtgtccgggatctcgacgct
ctcccttatgcgactcctgcattaggaagcagcccagtagtaggttgaggccgttgagcaccgccgccgc
aaggaatggtgcatgcaaggagatggcgcccaacagtcccccggccacggggcctgccaccatacccacg
ccgaaacaagcgctcatgagcccgaagtggcgagcccgatcttccccatcggtgatgtcggcgatatagg
cgccagcaaccgcacctgtggcgccggtgatgccggccacgatgcgtccggcgtagaggatcgagatctc
gatcccgcgaaattaatacgactcactataggggaattgtgagcggataacaattcccctctagaaataa
ttttgtttaactttaagaaggagatataccatgggcagcagccatcatcatcatcatcacagcagcggcc
tggtgccgcgcggcagccatATGGTAGTCAAATTCACTGACAGCCAAATCCAACATCTGATGGAGTATGG
TGATAATGATTGGAGCGAGGCAGAATTTGAGGACGCTGCTGCTCGTGATAAAGAGTTTTCAAGCCAATTC
TCCAAGTTGAAGAGTGCGAACGACAAAGGATTGAAAGACGTCATTGCGAACCCGCGTAATGACCTGACCG
ACCTTGAAAATAAGATTCGTGAGAAACTTGCTGCACGCGGTTTCATCGAAGTGCATACGCCTATTTTTGT
ATCTAAGAGTGCATTAGCCAAGATGACAATCACCGAGGATCATCCTTTATTCAAGCAGGTCTTCTGGATC
GACGACAAACGTGCCTTGCGTCCAATGCATGCGATGAATCTTGCTAAGGTACTGCGCGAGTTGCGCGATC
ACACAAAGGGACCAGTCAAGATCTTCGAGATTGGCTCGTGCTTCCGCAAGGAAAGCAAGTCATCGACGCA
TTTGGAAGAATTCACTATGCTGAACTTAGTTGAGATGGGACCCGATGGCGACCCTATGGAGCACCTTAAG
ATGTATATTGGAGACATCATGGACGCGGTTGGTGTAGAATACACCACCTCACGTGAGGAGTCTGATGTGT
ACGTAGAGACACTTGACGTGGAGATCAATGGAACTGAAGTTGCGTCAGGAGCAGTAGGTCCTCATAAGCT
TGACCCTGCCCACGATGTGCATGAACCCTGGGCAGGAATCGGATTCGGACTGGAGCGTCTGTTGATGCTT
AAGAACGGTAAATCGAATGCTCGTAAGACAGGCAAAAGTATCACCTATTTGAATGGTTACAAATTGGATT
AAggatccgaattcgagctccgtcgacaagcttgcggccgcactcgagcaccaccaccaccaccactgag
atccggctgctaacaaagcccgaaaggaagctgagttggctgctgccaccgctgagcaataactagcata
accccttggggcctctaaacgggtcttgaggggttttttgctgaaaggaggaactatatccggat
